# DPRM: DeBERTa-based potential relationship multi-headed self-attention joint extraction model

**DOI:** 10.1371/journal.pone.0329120

**Published:** 2025-08-05

**Authors:** Songjiang Li, Jinming Cao, Jiao Yang, Yunjiangcan He, Peng Wang

**Affiliations:** 1 College of Computer Science and Technology, Changchun University of Science and Technology, Changchun, China; 2 Chongqing Research Institute, Changchun University of Science and Technolo-gy, Chongqing, China; Zhejiang Normal University, CHINA

## Abstract

Traditional entity-relationship joint extraction models are typically designed to address generic domain data, which limits their effectiveness when applied to domain-specific applications such as manufacturing. This study presents the DeBERTa-based Potential Relationship Multi-Headed Self-Attention Joint Extraction Model (DPRM), which has been specifically designed to enhance the accuracy and efficiency of entity-relationship extraction in manufacturing knowledge graphs. The model is comprised of three core components: a semantic representation module, a relationship extraction and entity recognition module, and a global entity pairing module. In the semantic representation module, a DeBERTa encoder is employed to train the input sentences, thereby generating word embeddings. The capture of word dependencies is achieved through the utilization of Bi-GRU and Multi-Headed Self-Attention mechanisms, which serve to enhance the overall representation of the sentence. The relationship extraction and entity recognition module is responsible for identifying potential relationships within the sentences and integrating a relational gated mechanism to minimize the interference of irrelevant information during the entity recognition process. The global entity pairing module simplifies the model’s architecture by extracting potential relationships and constructing a matrix of global pairing entity pairs based on fault-specific data. The efficacy of the proposed model is validated through experiments conducted on fault datasets. The results demonstrate that the DPRM achieves superior performance, with an F1 score that surpasses that of existing models, thereby highlighting its effectiveness in the fault domain.

## 1 Introduction

In recent years, the convergence of information technology and manufacturing has emerged as a major trend in the global manufacturing sector. With the advent of the “Internet Plus” era, industrial intelligence has become central to the transformation and modernisation of manufacturing processes. A critical aspect of this transformation is the development of knowledge bases that integrate manufacturing data to enable more intelligent decision making [1]. This shift has sparked considerable interest in the creation of specialised domains for building knowledge graphs, which have the potential to revolutionise the use of manufacturing data [2,3].

At the core of building a knowledge base is knowledge extraction [4,5], a process that involves both entity recognition [6–8] and relationship extraction [9,10]. In the context of fault management, much of the available data is either unstructured or semi-structured, which poses a challenge for effective analysis. The low-value density of fault data, coupled with the strong coupling of key maintenance knowledge, hinders efficient data management and utilization [11]. Given the wealth of fault correlation information embedded in this data, advanced entity and relationship extraction techniques are required.

Traditional methods for knowledge extraction in the manufacturing default domain typically follow a pipeline approach [12–14]. First, fault-related entities are obtained through named entity recognition. Then, relationship extraction is performed among the candidate entities based on predefined relationship types. Although the pipeline model has high flexibility, it ignores the lack of interaction between two subtasks and the model cannot use the associated task information in it. In addition, there is a large amount of redundancy in the entity pairs generated by NER and not all pairs have relationships. For example, D et al. [15] used the pipelined approach to construct a triad for the fault domain of mine hoists, but the redundancy value of entity pairs in it was high. This redundancy and dependency makes the results of relational extraction heavily dependent on the accuracy of entity extraction, which can easily lead to the error propagation problem. To address these limitations, a joint entity and relationship extraction model has been proposed. This approach, which combines both tasks into a single model, mitigates the problem of error propagation and improves the accuracy of knowledge extraction. Entity-relationship joint extraction is a crucial subtask in information extraction and plays a fundamental role in the construction of knowledge graphs [16].

The application of joint extraction models to automatically generate fault knowledge graphs from fault data has significant potential for advancing intelligent maintenance and diagnostic systems [17]. Further research into the methods and techniques for constructing manufacturing knowledge graphs is essential not only for the continued innovation-driven growth of China’s manufacturing industry, but also for the global advancement of manufacturing technologies and economic development [18,19].

Zheng et al. [20] used sequence annotation to achieve joint extraction of entities and relations but overlooked the challenge posed by overlapping ternary relations. Similarly, Wei et al. [21] introduced a novel annotation framework but failed to address the issue of nested entities. The TPLinker model [22] framed the joint extraction task as a labeled pairwise connectivity problem, effectively solving both the relationship overlap and exposure bias problems. Although the above models perform well in general-purpose domains, they have significant limitations when dealing with manufacturing fault data, including limited ability to handle abbreviations and jargon, insufficient handling of nested entity structures, weak capture of contextual dependencies, sensitivity to uneven distribution of relational labels, and insufficient adaptability to dynamic data. To address this gap, we propose a joint extraction model based on DeBERTa with a latent relational multi-headed self-attention mechanism. This model is designed to provide a more comprehensive solution to the unique challenges posed by manufacturing fault data, ultimately improving the accuracy of the extracted triples.

The main contributions of this thesis are as follows:

This paper introduces a novel semantic representation module, DeBERTa-Bi-GRU-Multi-Headed Self-Attention (DBM). The incorporation of DeBERTa [23] and Multi-Headed Self-Attention [24] into the semantic representation process enables this module to enhance the joint extraction model’s capacity to address ambiguity at the entity boundaries. DeBERTa is fine-tuned for specific tasks during training, thereby mitigating the issue of entity boundary ambiguity. The Multi-Headed Self-Attention mechanism enhances sentence-level representation by capturing intricate inter-word semantic dependencies, thereby improving model flexibility and performance.

Furthermore, we introduce a relational gated mechanism into the relationship extraction and entity recognition module. This mechanism enables the model to more effectively retain domain-specific relationship information, thereby enhancing the sensitivity of relationship extraction. This approach addresses the issues of relationship redundancy and overlaps, thereby improving the precision of the extraction process.

This represents the first time that a method based on the global pairing of entity pairs within a potential relationship matrix has been proposed in the field of manufacturing fault analysis. This approach simplifies the model’s complex architecture by reducing irrelevant relationship predictions, thereby significantly improving the accuracy of extracted triples.

Experiments were conducted on a manufacturing automobile failure dataset provided by a competition, and the results show that the present model outperforms several other state-of-the-art models in terms of recall followed by precision, and maintains a comparable level of performance under a specific domain.

## 2 Related work

As the manufacturing industry continues to generate massive amounts of data, extracting entity relationships from fault data is critical to laying the groundwork for building fault knowledge graphs. These graphs enable intelligent maintenance, real-time fault diagnosis, and overhaul strategies in high-end equipment manufacturing. However, research in the area of manufacturing fault data remains sparse, and significant limitations remain, particularly with respect to the availability and scope of data specific to manufacturing fault scenarios.

In the context of domain-specific knowledge graph construction. Many scholars have researched domain-specific data [3,25–29] Yu et al. [30] proposed a relationship extraction method designed to build domain-specific knowledge graphs. Their approach involves extracting superior-subordinate relationships from web encyclopedia classification systems, while non-superior relationships are derived from unstructured text using a convolutional residual network optimized with an improved cross-entropy loss function. However, convolutional neural networks and residual networks typically require significant computational resources, which can limit the applicability of this method in resource-constrained environments. This is because real-time processing of fault data is often required in these environments.

Zhu et al [31] developed a joint model, ITIRel, for extracting Internet of Things (IoT) threat intelligence (ITI) entities and relationships. By incorporating domain-specific knowledge, their model effectively learns domain-related terms, leading to improved accuracy in relationship extraction. In fact, the model is designed specifically for IoT threat intelligence and does not take into account the unique challenges in manufacturing fault data, such as the large number of abbreviated fault names and the presence of technical jargon. Xu et al [32] introduced a novel relationship extraction model, GraphJoint, which frames the task as mapping relationships to entities. While GraphJoint addresses challenges such as overlapping entity pairs within multiple relationships and overlapping subjects and objects in a relationship, it is primarily designed for social media texts, which differ significantly from manufacturing fault data in terms of language style and context dependency. For example, social media texts often contain informal language and short sentences, whereas manufacturing fault data is highly technical and context-dependent.

Wang et al [33] proposed a deep learning-based method for automatically extracting and representing relationships in the domain of fall protection requirements and successfully extracting domain-specific relationships. The performance of the model is limited to its specific domain and does not generalize well to manufacturing fault data, where relationships and entities are more complex and context-dependent. Sui et al [34] introduced a non-autoregressive parallel solver network based on transformers, as opposed to traditional autoregressive methods that generate triples sequentially. Their model is capable of outputting relational triples in parallel, thus addressing the problem of ensemble prediction. Although the model performs well on specific datasets, it generalizes poorly when dealing with manufacturing fault data, which has a highly uneven distribution of relational labels. Although the model performs well on specific datasets, it has poor generalization ability when dealing with manufacturing fault data because the distribution of relational labels in manufacturing fault data is extremely uneven.

To address the challenge of overlapping relations in text, Wei et al. [21] proposed a framework that treats relations as mapping functions from head entities to tail entities, effectively solving the problem of overlapping relations. Nevertheless, the model does not consider the nested entity structure that is common in manufacturing fault data, which limits applicability to other domains. Xu et al. [35] introduced the BERT-PAGG model for relation extraction, which incorporates entity position information and combines local features extracted by the PAGG module with entity vector representations from BERT. While the model captures valid semantic features through the attention mechanism, its embedding model is relatively homogeneous, limiting its ability to deal with diverse and domain-specific terminology in manufacturing fault data. Tao et al. [36] put forth a joint extraction model based on multiple-headed attention mechanisms and graphs (MAGCN). The model constructs an adjacency matrix using attention and combines it with a graph convolutional network (GCN) to achieve joint extraction, thereby circumventing errors that might otherwise result from external dependency analysis. Nonetheless, the introduction of irrelevant relational information into the model may reduce the model’s sensitivity to key relationships, which is particularly critical in manufacturing fault data and can affect the accuracy of fault diagnosis. Zhao et al. [37] put forth a joint entity and relationship extraction model based on a directed relation graph attention network. By constructing a dictionary of Chinese relational cue words. This approach facilitates enhanced interaction between entities and relations, thereby improving the accuracy of both entity and relationship extraction. However, the model’s reliance on predefined dictionaries limits its adaptability to emerging or changing manufacturing fault scenarios where new terms and relationships may be updated frequently.

For feature extraction capability, Xue et al. [38] put forth a combined extraction model based on a multi-head attentional neural network. The model enhances feature extraction; however, it does not account for the entity boundary ambiguity problem, thereby limiting its ability to fully enhance sentence representations. Yuan et al. [39] proposed a solution to the redundant operation exist in the approach of performing entity recognition first, followed by relation detection for each possible entity pair. They introduced a relation-specific attention network (RSAN) to address this issue. However, the model fails to consider the impact of potential relationships on entities and does not address the uneven distribution of relationship labels. These are the key challenges that exist in manufacturing fault data. Lai et al. [40] put forth the RMAN model for joint entity and relationship extraction, which comprises a multi-feature fusion encoder for sentence representation and a sequence labeling decoder. However, the model’s failure to consider inter-word semantic information results in an incomplete enhancement of sentence representations. This is especially critical in manufacturing fault data, where contextual information is critical for accurate extraction of faulty entities.

In order to solve problems such as entity nesting, Zhang et al [41] proposed MGBERT-Pointer, a method that combines multi-granularity BERT adapters and efficient global pointers for enhancing Chinese-named entity recognition. The method enhances the model’s ability to deal with complex contexts by integrating multiple adapters and solves the boundary recognition problem by using rotated positional embedding, which effectively improves the recognition performance of nested entity structures. However, the model relies on existing scenarios and requirements, and once these scenarios and requirements change, the model may no longer be applicable. This lack of adaptability is particularly acute in dynamic manufacturing fault data environments.

## 3 Methodology

**Fig 1:**
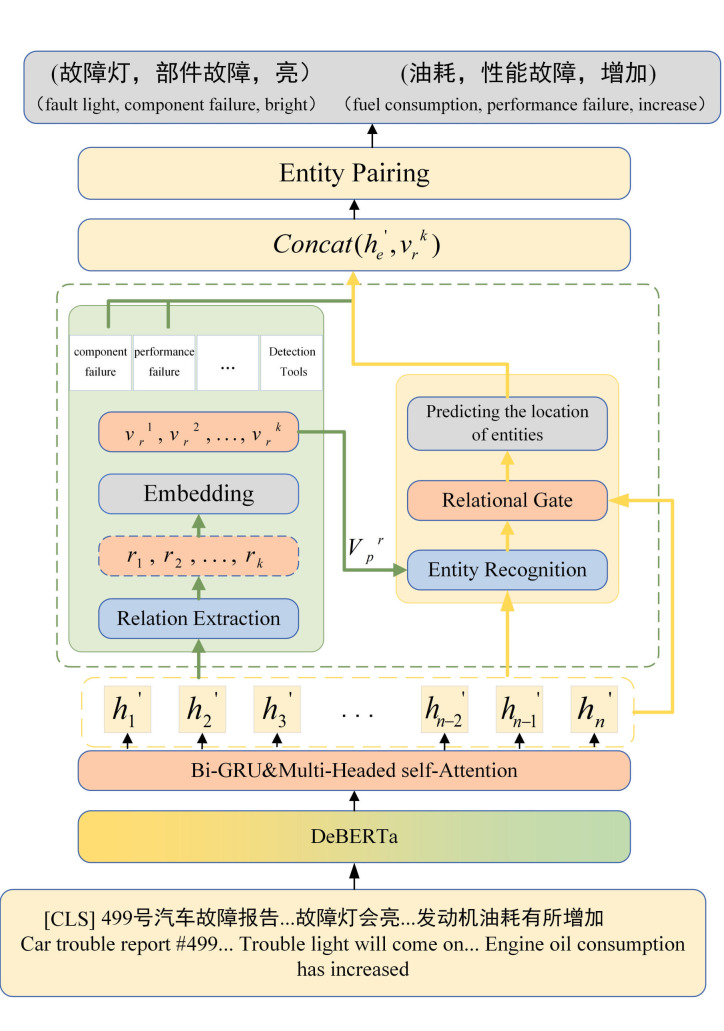
Model structure.

In this paper, we put forth a joint entity-relationship extraction model, DPRM. Fig 1 depicts the architecture of our proposed model, which encompasses three principal modules: the semantic representation module, the relationship extraction and entity recognition module, and the global entity pairing module. In the semantic representation module, the DeBERTa encoder is employed to pre-train the input sentences, followed by the application of Multi-Headed Self-Attention atop a Bi-GRU network to enhance sentence encoding. In the relationship extraction and entity recognition module, we integrate an attention mechanism based on potential relationships with a relational gated mechanism, thereby enabling the joint extraction of relationships and recognition of entities within sentences. Ultimately, the global entity pairing module generates a global pairing entity Pair Matrix, which models all entity pairs across all relations within the sentence, thereby producing the final relational triples. The following sections provide a detailed description of each module.

### 3.1 Task definition

Given a sentence $W=(w_{1},w_{2},...,w_{n})$ , Relationship to predefined $R=\{r_{1},r_{2},...,r_{k}\}$, where n denotes the length of the sentence and k denotes the number of relations. In this paper, we set the ternary of the knowledge graph to be G={(s,r,o)|s,o∈E,r∈R}, where E is the set of entities, s, o denotes the head and tail entities, and r denotes the relationship corresponding to the head and tail entities. The key symbols used in this chapter and their meanings are shown in Table 1 below:

**Table 1:** Symbols and meanings.

Symbols	Meaning
$W=(w_{1},w_{2},...,w_{n})$	Sentences
$X=(x_{1},x_{2},...,x_{n})$	Sentence encoding embedding representation
Avgpool	Average pooling operation
$P_{r}$	Potential relationship probability distribution
$Loss_{r}$	Model loss
${V_{p}}^{r}$	Set of potential relationship vectors
$P^{k}$	Pairing probabilities for different entity pairs
E	Entity Set
$\theta_{e}$	Thresholds
$R_{p}$	Number of potential relationships in a sentence
$e_{\max}$	Maximum total number of entity collections

### 3.2 Semantic representation module

**Fig 2:**
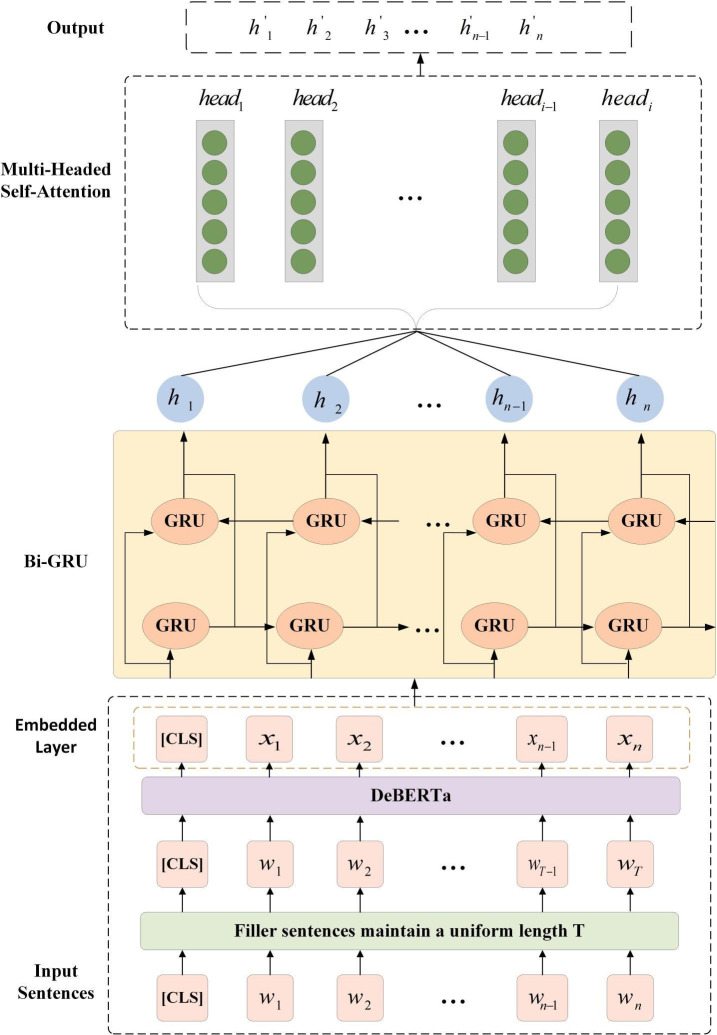
The overall framework of DBM.

This paper introduces a semantic representation module, DeBERTa-Bi-GRU-Multi-Headed Self-Attention (DBM). The DBM model is comprised of three key components: the pre-trained DeBERTa model, Bi-GRU, and Multi-Headed Self-Attention. This module addresses the issue of entity boundary errors in fault data while fully enhancing sentence representations to more effectively capture the semantic relationships between words, thereby reducing the impact on the subsequent enhanced sentence decoding. The overall framework of DBM is depicted in Fig 2.

#### 3.2.1 Embedding.

First given a sentence $W=(w_{1},w_{2},...,w_{n})$, The length of the sentence is n, The faulty sentences are then filled in so that the sentences maintain a uniform length T, Subsequently, a pre-trained model(DeBERTa) is utilized as an encoder to capture the embedded representation of each token $X=(x_{1},x_{2},...,x_{n})$, where n denotes the sequence length, as shown in formula (1):


$$X=DeBERTa(w_{1},w_{2},...,w_{T})$$ (1)

$w_{T}$ denotes the input representation of each token, $x_{i}\in R^{d}$, d is the dimension of the word embedding. The integration of the pre-trained DeBERTa encoder into the task improves the model’s classification accuracy and enhances the semantic representation of the data. This is of paramount importance for the accurate analysis of automotive fault data.

#### 3.2.2 BiGRU+multi-headed self-attention.

Following the processing of the word embeddings, the word embedding $X=(x_{1},x_{2},...,x_{n})$ is input into the Bi-Gated Recurrent Unit (Bi-GRU) [42] in order to capture the complex dependencies between words. Bi-GRU is an improved Recurrent Neural Network (RNN) that processes sequence data by introducing two separate GRU layers: one for forward sequences and another for reverse sequences. This structure allows the model to capture the forward and backward dependencies in the sequences. In the context of fault data, the Bi-GRU is an effective method for identifying dependencies between variables, as illustrated in Fig 1. For example, it can discern the relationship between “fault light” and “bright,” as well as “fuel consumption” and “increase.” The Bi-GRU not only provides accurate evaluations based on training parameters but also exhibits significant advantages in training speed and modeling efficiency compared to the traditional Bi-LSTM.

The output of Bi-GRU as shown in formula (5):


$$z_{t}=\sigma (W_{z}\cdot [h_{t-1},x_{t}])$$ (2)

$$r_{t}=\sigma (W_{r}\cdot [h_{t-1},x_{t}])$$ (3)

$${\tilde{h}}_{t}=\tanh(W\cdot [r_{t}*h_{t-1},x_{t}])$$ (4)

$$\vec{h_{t}}=((1-z_{t})*h_{t-1}+z_{t}*{\tilde{h}}_{t}$$ (5)

In this context, the symbol $z_{t}$ represents the update gate, the letter $r_{t}$ denotes the reset gate, ${\tilde{h}}_{t}$ refers to the candidate activation, $h_{t-1}$ indicates the output of the previous unit, $\vec{h_{t}}$ is the output of the current unit, $W,W_{z},W_{r}$ is the parameter, σ denotes the Sigmoid function, and the inverse output can be obtained similarly from Eqs. (2)-(5), where $h_{t-1}$ can be replaced by $h_{t+1}$, which represents the output of the future cell, connects the forward and backward hidden state outputs of the Bi-GRU, serving as the initial context representation. As shown in formula (6):


$$h_{t}=[\vec{h_{t}};\overset{\leftarrow }{h_{t}}]$$ (6)

The output of Bi-GRU is $h=(h_{1},h_{2},...,h_{n})$, where $h_{n}\in R^{2\times d_{h}}$, $d_{h}$ represents the dimensions of the hidden states in the Bi-GRU. The Bi-GRU not only offers precise assessments of each word in a fault sentence but also effectively captures the intricate interdependencies between words.

To further enhance the feature representation of the sentence, the output layer of the Bi-GRU is employed as the input to the Multi-Headed Self-Attention mechanism. In a sentence indicating a malfunction, the attention heads may focus on “fault light,” “bright,” “engine,” “fuel consumption,” and “increase.” This parallel processing enables the model to comprehend the sentence from multiple perspectives concurrently, thereby enhancing the overall sentence representation. The following equation provides the formula for the Multi-Headed Self-Attention layer:


$$Attention(Q,K,V)=soft\max(\frac{QK^{T}}{\sqrt{d_{k}}})V$$ (7)

$$head_{i}=Attention(Q{W_{i}}^{Q},K{W_{i}}^{K},V{W_{i}}^{V})$$ (8)

$$M=Concat(head_{1},head_{2},...,head_{i})W^{O}$$ (9)

The Q, K, and V represent the queries, keys, and values of the input sequences, respectively. $d_{k}$ denotes the dimension of Q and K. ${W_{i}}^{Q}$, ${W_{i}}^{K}$, ${W_{i}}^{V}$, $W^{O}$ represents the weight matrix, the softmax function is used to normalise the attention score. The attention score in equation (7) determines the importance of each position to the other positions. Eq. (8) represents how each attention head is calculated,. With multiple attention heads, the model can capture information from different subspaces in the sequence, and finally Eq. (9) by computing multiple attention heads in parallel and stitching the results together, we denote the output of the multi-head attention layer as $h^{\prime }$. The sentence is represented as $H=\{{h^{\prime }}_{1},{h^{\prime }}_{2},...,{h^{\prime }}_{n}\}$.

### 3.3 Relation extraction and entity recognition module

**Fig 3:**
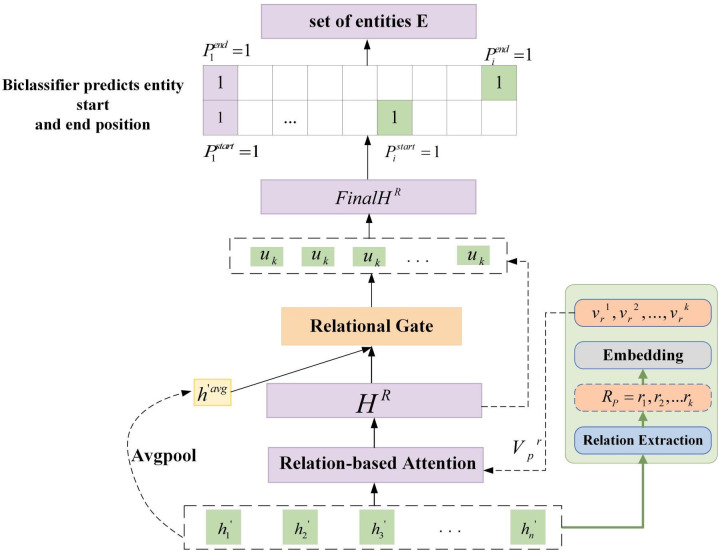
Fusion relational gated mechanism module diagram.

We put forth an attention mechanism for identifying potential relations, integrated with relational gated mechanism for fault data. The latent relational attention mechanism attention the model’s attention towards information that is relevant to the presence of latent fault relations within the sentence. The incorporation of the Relational Gated Mechanism serves to mitigate the interference of less relevant relational information. Although the latent relational attention mechanism may introduce some less relevant information, the relational gating mechanism enables the model to selectively disregard such information. The combination of the latent relational attention mechanism with the relational gating mechanism enhances the model’s capacity to capture information about entity relations within a sentence, thereby improving its expressive power. The specific structure is shown in Fig 3.

#### 3.3.1 Relation extraction.

After semantic representation, the vector representation H of the sentence is obtained. As shown in Fig 3, the set of potential relations {component failure, performance failure} is extracted from the sentence based on H. In the context of manufacturing fault data, each sentence may contain multiple relationships. To address this, we adopt a multi-label classification strategy to identify all potential relationships present in the sentence. The processed sentence representation h is then projected into the relationship detection space for multi-label classification. The specific implementation process is in the following formula:


$${h^{\prime }}^{avg}=Avgpool(h^{\prime })$$ (10)

$$P_{r}=\sigma (W_{r}{h^{\prime }}^{avg}+b_{r})$$ (11)

AvgPool represents the average pooling operation, ${h^{\prime }}^{avg}\in R^{d_{e}}$ denotes the sentence vector, and $d_{e}$ refers to the dimensionality of the tokens output by the model. $P_{r}$ represents the probability distribution of all potential relations contained within a sentence after applying the multi-label classification strategy. $W_{r}\in R^{d_{e}\times 1}$, $b_{r}$ are the trainable weights and biases.

We denote the set of relations detected by equation (11) as $R_{P}$. To evaluate the discrepancy between the relationship labels predicted by the model and the actual labels, we minimize the binary cross-entropy loss function. The corresponding formula is as follows:


$$Loss_{r}=-\frac{1}{n}\sum_{i=1}^{R}\left(y_{i}\log P_{r}+(1-y_{i})\log(1-P_{r})\right)$$ (12)

where $y_{i}\in \{0,1\}$ indicates whether the current relation label belongs to the relation label present in the sentence. r is the number of elements in the relation set.

#### 3.3.2 Entity recognition.

As shown in Fig 3, we encode the set of potential relations extracted from the sentence and perform an attention computation with the sentence representation H, yielding relation-aware attention weights that the generation of the sentence representation $H^{R}$. To account for the presence of fault data and to improve the extraction of entities from the sentence, we introduce a fusion relational gated mechanism. This mechanism exploits potential relational information to improve the performance of the attention mechanism and is more meaningful for subsequent entity recognition compared to the original sentence. The set of all potential relations present in a sentence can be obtained by relation extraction $R_{P}$.Suppose the maximum number of relations present in the sentence is $r_{\max}$, and $r_{\max}$ is less than or equal to the predefined total number of relations R, The potential relationships are encoded by a relationship encoder to obtain potential relation vectors V. The resulting potential relation vectors V are then used for attention computation with the sentence vectors H, computing relational attention weights between each word in the sentence and the corresponding relational embedding. The computational procedure is formalized in the following formula:


$$Q=W_{query}H+b_{query}$$ (13)

$$K=W_{key}{V_{p}}^{r}+b_{key}$$ (14)

$$R_{H}=soft\max(\frac{QK^{T}}{\sqrt{d_{k}}}){V_{p}}^{r}$$ (15)

$$H^{R}=W_{H\_R}Concat(H,R_{H})+b_{H\_R}$$ (16)

Where $W_{query},W_{key}\in R^{d_{e}\times d_{k}},W_{H\_R}\in R^{d_{e}\times 2d_{k}}$。${V_{p}}^{r}\in R^{r_{\max}\times d_{e}}$ is the trainable embedding of the relations in the sentence, $H^{R}\in R^{n\times d_{e}}$ is the sentence representations that incorporate relevant relations information.

So far, we have obtained sentence representations containing specific relations, which are meaningful for subsequent entity extraction only when the relation is positive to the sentence, and the representation oriented towards irrelevant relations will only confuse the subsequent entity extraction process, in order to adaptively control the relation information provided by the previous attention layer, the relation gating mechanism is chosen as a bridge, and the specific implementation process is shown in the following equation:


$$g_{k}=\sigma ((W_{1}{h^{\prime }}^{avg}+b_{1})⊕(W_{2}H^{R}+b_{2}))$$ (17)

$$u_{k}=g_{k}⊙\tanh(W_{3}H^{R}+b_{3})$$ (18)

$$FinalH^{R}=H^{R}⊙u_{k}$$ (19)

Where ${h^{\prime }}^{avg}$ denotes the initial raw sentence representation and $H^{R}$ is the sentence representation containing information about potential relationships. $W_{1},W_{2},b_{1},b_{2},W_{3},b_{3}$ is a model parameter, ⊕ denotes a join operation, ⊙ denotes an element multiplication, and σ is a sigmoid function. The function returns values from 0 to 1 so that the result can be considered as a percentage of the information to be retained, Eq. 17 aims to measure which of the initial raw sentence representation ${h^{\prime }}^{avg}$ and the sentence representation $H^{R}$ containing potential relational information is more useful for entity extraction. The gating information $g_{k}$ combines the original sentence representation and the sentence representation containing the potential relational information for controlling the flow of information. $u_{k}$ is the retained relational feature, and finally $u_{k}$ is multiplied by $H^{R}$ to obtain the final representation $FinalH^{R}$. The final representation $FinalH^{R}$ is the one that is used to control the flow of information.

After obtaining the representation of the relationally fused sentence information, two binary classifiers are used to independently predict the start and end positions of entities, thereby increasing the model’s accuracy in entity recognition. The model does not distinguish between specific subject and object entities, which simplifies its extraction architecture. A simple 1/0 labeling scheme is used, where each label indicates whether a word is part of an entity. Specifically, $P_{start_{i}}=1$ if a word marks the beginning of an entity, and $P_{start_{i}}=0$ otherwise, as formalized in the following formula:


$${P_{i}}^{start}=\sigma (W_{start}{h_{i}}^{r}+b_{start})$$ (20)

$${P_{i}}^{end}=\sigma (W_{end}{h_{i}}^{r}+b_{end})$$ (21)

Where $W_{start},W_{end}\in R^{d_{e}}$ are trainable weights, ${P_{i}}^{start}$ and ${P_{i}}^{end}$ represent the probability that the i-th token in the sentence is the start or end of the entity, respectively. The formula is shown below:


$$Loss_{e}=-\frac{1}{n}(\log{P_{\theta }}^{start}(s|H^{R})+\log{P_{\theta }}^{end}(s|H^{R}))$$ (22)

where $P_{\theta }(s|H^{R})=\prod {\mathrm{\backslash nolimits}}_{i=1}^{n}{p_{i}}^{\left\{y_{i}=1\right\}}(1-p_{i}{)}^{\left\{y_{i}=0\right\}}$ denotes the i-th token in the sentence.

### 3.4 Global entity pairing module

Building on previous work, we have obtained the set of potential relationships and the set of entities within the sentence. Since different relationships exist between different pairs of entities, they form ternary groups. To mitigate the problem of multiple entity overlap, we first compute the sequence of entity set vectors He by combining the sentence representation vector H with the entity set E. Next, the entity set vectors are combined with each potential relationship vector. Using an affine transformation, we construct a matching matrix that identifies entity pairs under specific relationships within the fault data in the manufacturing domain. This approach allows the ternary group to be obtained in one step using only potential relationships within the matrix, which significantly reduces both the space and time complexity of model training.

**Fig 4:**
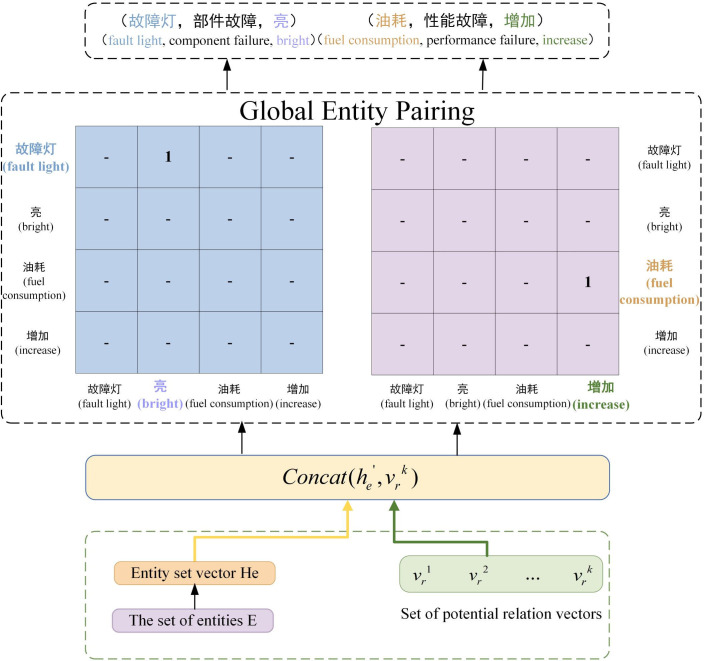
Global entity pairing process.


The specific pairing process is illustrated in Fig 4. First, all entities present in the entity set are identified. Using the sentence-based representation vector H, the corresponding set vector of identified entities is obtained. Then, an affine transformation method is applied to construct the entity pairing matrix for each potential relationship. The scores of entity pairs within the matrix are then evaluated, and any pair with a score above a threshold $\theta_{e}$ is retained, while those below the threshold are filtered out. For example, consider the ternary groups “fault light, component failure, bright” and “fuel consumption, performance failure, increase”. Following the outlined steps, we first identify the entity set “fault light, bright, fuel consumption, increase”. Using the representation vector H and the entity set, we then compute the entity set vector sequence He, followed by the construction of the entity matching matrix for each potential relationship. This process results in the formation of two triples, as formalized in the following formula:

$${h^{\prime }}_{e}=\frac{{h^{\prime }}^{start}+{h^{\prime }}^{end}}{2}$$ (23)

$$h^{\prime }er=concat({h^{\prime }}_{e},{v_{r}}^{k})$$ (24)

$$P^{k}=\sigma (W_{e\_r}[h^{\prime }erW_{e}h^{\prime }er]+b_{e\_r})$$ (25)

where $W_{e\_r},W_{e}\in R^{d_{e}\times d_{e}}$ are trainable weights, ${v_{r}}^{k}\in R^{1\times d_{e}}$ denotes the trainable embedding vector, and $P^{k}$ is the probability of pairing different entity pairs directly. We employ a biaffine model over the sentence to create a l×l scoring tensor, where l represents the maximum number of entities in the set of sentence entities. The sigmoid function is subsequently used to determine whether the entity pairs at the identified positions satisfy the pairing criteria. The model is trained by minimizing the binary cross-entropy loss function, the formula is shown below:


$$Loss_{g}=-\frac{1}{n}\sum_{k=1}^{R_{p}}\sum_{i=1}^{e_{\max}}\sum_{j=1}^{e_{\max}}{y^{k}}_{i,j}\log({P_{i,j}}^{k})+(1-{y_{i,j}}^{k})\log(1-{P_{i,j}}^{k})$$ (26)

where $R_{p}$ represents the number of potential relationships in the sentence and $e_{\max}$ represents the maximum total number of entities in the set, the model as a whole was trained jointly and the total loss of the model was obtained as shown in the following formula:


$$Loss=\alpha Loss_{r}+\beta Loss_{e}+\gamma Loss_{g}$$ (27)

where α, β and γ are custom constants. In our experiment, we set α=β=γ=1.

## 4 Experiments

In this section, we detail the fault dataset as well as the details of the experiment and analyze the experimental section results.

### 4.1 Experiment settings

#### 4.1.1 Datasets.

This study employs data from the high-end equipment manufacturing knowledge graph automated construction contest, provided by CCF BDCI [17], in conjunction with external open-source data from CCL [43]. The fault dataset is semi-structured, comprising records written by business experts or professional maintenance personnel that detail relevant equipment anomalies and fault troubleshooting procedures. Each record comprises a description of the fault phenomenon, its causes, solutions, and the troubleshooting process. Collating from data sets, a total of 6118 fault records were obtained, comprising 4491 training samples, 830 test samples, and 797 validation samples. Table 2 presents the distribution of relationship types within the dataset.

**Table 2:** Relationship type distribution statistics.

Relationship type	Quantities
Component failure	8825
Detection tools	28
Performance failure	630
Constitute	218

Moreover, to assess the model’s capacity to process ternary groups, the training set is classified into three categories. The categories are defined as follows: normal, EPO, and SEO. Additionally, the number of ternary groups present in each category is presented in Table 3.

**Table 3:** Analysis of data set details.

Normal	SEO	EPO	N = 1	N = 2	N = 3	N = 4	N>=5
4765	5536	16	1693	663	309	186	562

#### 4.1.2 Implementation details.

For our experiments, we used a computer equipped with an Intel Core i9-13900K processor, NVIDIA GeForce RTX 4090 GPU*1 (with 24GB GDDR6 memory), and 128GB DDR5 RAM. The operating system is Ubuntu22.04, we use Python3.8 for programming and pipe our environment through Anaconda, which contains cuda11.1, and pytorch1.8.During the training process, DeBERTa uses the V3 version, the maximum length of the sentence is set to 128, the Bi-GRU follows the Relationship embedding dimension is set to 300, the Multi-Headed Self-Attention dimension is set to 600, and the number of heads is 8. We also apply the dropout mechanism and set the rate to 0.3. We tuned our model on the effective set to adjust important hyperparameters. For our own module, given the size of the dataset, we set batch to 24, and the training parameters on the faulty dataset are given in Table 4.

**Table 4:** Hyper parameter.

Hyper-parameters	Values
Maximum sentence length	128
Word embedding dimension	100
Relationship embedding dimension	300
Bi-GRU dimension	300
Multi-Headed Self-Attention dimension	600
Dropout	0.3
Learning rate	0.001
Entity threshold	0.9
Relationship threshold	0.9
Entity pair thresholds	0.5
Batch Size	24
Epoch	60

#### 4.1.3 Computational efficiency.

In this study, we use a unified configuration as shown in Table 4 to compare the performance of the models. We choose three models, TPLinker, RMAN and ERGM, to compare with the DPRM model proposed in this study. The TPLinker model has a high computational complexity $O\left(kn^{2}\right)$ by iterating through all the token pairs and labeling the token links using three matrices to identify the relations, where k is the size of the relation set and *n* is the length of the input sentence. The RMAN model has a complexity of O(n·(1+h+k)), where *h* is the number of heads of the multi-head attention mechanism. The complexity of the RMAN model is linearly related to the input sentence length *n* and the relation set size k, and is affected by the number of heads of multi-head attention, *h*. The ERGM model uses entity matching matrices to identify the entities in the sentence, and thus has a computational complexity of $O\left(n^{2}+e^{2}\right)$, where *e* is the number of entities in the sentence. The complexity of the DPRM model proposed in this study is $O\left(nd+nk+e^{2}\right)$, where *d* is the dimension of the DeBERTa model, and *e* is usually much smaller than *n*. Therefore, the DPRM model has a significant advantage in terms of computational complexity compared to the other models.

In addition, the DPRM model outperforms the other models in terms of training time. TPLinker needs to process all the relations, which greatly increases the time cost of training and inference. Compared to the RMAN model, the DPRM model does not need to identify the specific types of entities, which simplifies the model structure. The length of the labeled entity matching matrix constructed by ERGM is much higher than that of the DPRM model because the number of tokens in a sentence is more than the number of entities. Specifically, the training time per epoch for the DPRM model is 981 seconds compared to 1,134 seconds for ERGM, making DPRM 1.15 times faster than ERGM. In terms of memory occupation, the memory occupation of TPLinker, RMAN, ERGM, and DPRM models are 16 GB, 8 GB, 12 GB, and 6 GB, respectively. DPRM achieves the optimal results compared to other model architectures. These results fully demonstrate that the DPRM model combines the efficiency and performance of the model and will be more competitive in the real-world application scenarios of faulty datasets.

#### 4.1.4 Evaluation metrics.

In order to compare with previous modeling experiments, we use Precision (P), Recall (R) and F1 values to evaluate the model and thus serve as evaluation criteria.

#### 4.1.5 Baseline models.

We compared the model with the following seven models:

(1) Novel Tagging [20]: Transforming entity relationship extraction into a sequence labeling task does not address the problem of overlapping ternary relationships.
(2) CopyRE [44]: The Seq-to-Seq relationship extraction model, which uses a replication mechanism to realize the joint extraction of multiple triples, fails to solve the problem of fuzzy head and tail entity boundaries.
(3) CasRel [21]: A new labeling framework is proposed, which first proposes the head entity and recognizes the tail entity based on the given relationship type. However, the problem of entity nesting is not addressed.
(4) TPlinker [22]: Transforming the entity-relationship joint extraction framework into a character-pair linking problem effectively solves the relationship overlap problem and the exposure bias problem.
(5) RSAN [39]: A relation-specific attention network is proposed to solve the entity overlap problem.
(6) RMAN [40]: An XML model for joint extraction of entities and relations is proposed, which considers the semantic information between words and effectively solves the ternary overlapping problem
(7) ERGM [45]: Incorporating candidate relations into the relational attention mechanism simplifies the model structure by decomposing the joint extraction of entity relations into subtasks.

### 4.2 Experimental results and analysis

#### 4.2.1 Comparison with other models.

In this paper, an automobile fault text dataset is used for comparison experiments. As shown in Table 5, bold indicates the best results.

**Table 5:** Results of baseline models on fault datasets. Bold marks the best result.

Meodel	Precision(%)	Recall(%)	F1 (%)
Novel Tagging	0.393	0.282	0.381
CopyRE	0.498	0.432	0.475
CasRel	0.585	0.591	0.587
TPLinker	0.612	0.672	0.640
RSAN	0.783	0.763	0.771
RMAN	0.832	0.851	0.843
ERGM	0.856	0.864	0.862
**our**	**0.863**	**0.876**	**0.872**

The results demonstrate that the models proposed in this paper exhibit notable enhancements in terms of precision, recall, and F1 score. The our model was enhanced by incorporating DeBERTa and Multi-Headed Self-Attention into the semantic representation module, resulting in a 2.9% improvement in F1 score compared to RMAN. Furthermore, we introduce a relational gated mechanism within the entity recognition and relationship extraction module of ERGM, which effectively reduces the interference of irrelevant information. The proposed model demonstrates a 1.0% improvement in F1 score compared to ERGM, further substantiating the efficacy of the relational gated mechanism in mitigating the impact of irrelevant information on entity recognition tasks and enhancing entity recognition performance. Finally, the affine transformation method is utilized for the first time to construct the entity pairing matrix under specific relations in the manufacturing fault domain dataset, thereby significantly reducing the space and time complexity of model training.

Limitations: As can be seen from Table 6, the accuracy of the model does not reach the desired optimal value, which we can conclude after analysis, firstly, because the model uses the Chinese fault dataset, and a large number of abbreviated terminology is used in the dataset, such as component unit: EPC (abbreviation for lamp), ABS (control unit) and so on. The categories in the dataset are not balanced, as can be seen from Table 2, the number of relationship types for detection tools is much less than that for component failures, and the total number of relationship types for component failures is greater than the sum of the remaining three relationship types, which leads to the failure of the DPRM to fully and adequately learn the ternary with fewer relationship types on the dataset with unbalanced categories. Secondly, the limited amount of data in the fault dataset results in a small amount of data for training as well as testing the model. Finally, due to the above limitations, the DPRM model has limited ability to generalise to English datasets and out-of-domain data. It also has limited ability to parallelise computation on large-scale data. But, the DPRM model outperforms other models in significantly handling overlapping relationships and class imbalance problems in the fault domain. It is also more accurate than other existing models in handling faulty domain data.

**Table 6:** Precision values for the model on Normal, SEO, EPO.

Model	fault datasets		
	Normal	SEO	EPO
TPLinker	0.635	0.451	0.314
RMAN	0.841	0.773	0.676
ERGM	0.861	0.742	**0.768**
**our**	**0.866**	**0.787**	0.749

#### 4.2.2 Noise data testing and analysis.

**Fig 5:**
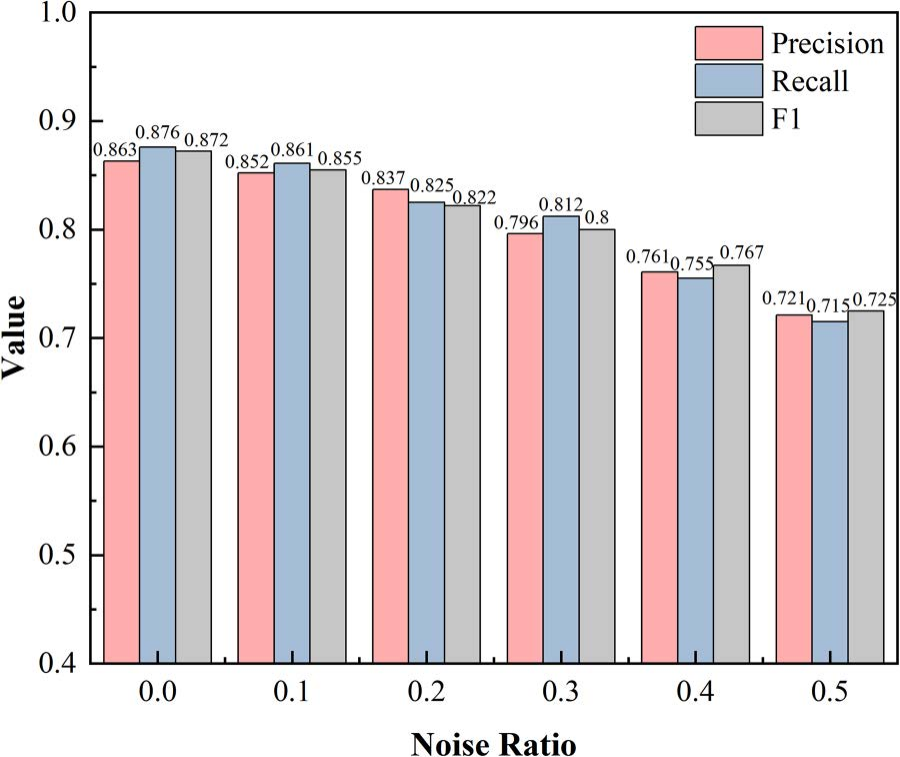
Performance comparison chart for different noises.

In order to evaluate the robustness of the model to noisy data, we introduced different percentages of noise in the dataset (noise percentage from 0% to 50%) and tested the variation of the model’s performance under different noise levels. The experimental results are shown in Fig 5 below:

The experimental results show that the performance of the model gradually decreases with the increase of the noise proportion, but it shows strong robustness to low noise data. When the noise proportion is 0%, the Precision, Recall, and F1 values of the model are 0.863, 0.876, and 0.872, respectively; when the noise proportion is increased to 20%, the F1 value still remains at 0.822, indicating that the model has a strong adaptive ability to low-noise data. However, as the noise percentage increases further, the model performance decreases faster, for example, the F1 value decreases to 0.725 when the noise percentage is 50%. As the noise percentage increases, the Recall decreases from 0.876 to 0.715. we analyze that the noise causes the model to miss some True Positives, which decreases the Recall. The experimental results show that the model can adapt to low-noise data. The results show that the model is robust to noisy data, but the performance of the model gradually decreases as the proportion of noise increases. At low noise level (0%−20%), the model’s performance decreases more slowly, indicating that it has strong adaptability to low noise data, and at medium-high noise level (20%−50%), the model’s performance decreases faster, indicating that its robustness to high noise data needs to be further improved. Through the above conclusions, we have analyzed that the DPRM model shows strong robustness in noisy data environments, and the DPRM firstly reduces the sensitivity to local noise by capturing global dependencies through the multi-head self-attention mechanism. Then, it dynamically filters low-confidence relational information through the relational gating mechanism to reduce the interference of noise on the entity recognition task. Finally, Dropout is used during the training process, thus preventing the model from overfitting the noisy data. In the future, we plan to balance the impact of noisy data by data cleaning and generating more high-quality samples through data enhancement techniques, thus further improving the model’s performance in high-noise environments.

#### 4.2.3 Analysis of AUC-PR results.

**Fig 6:**
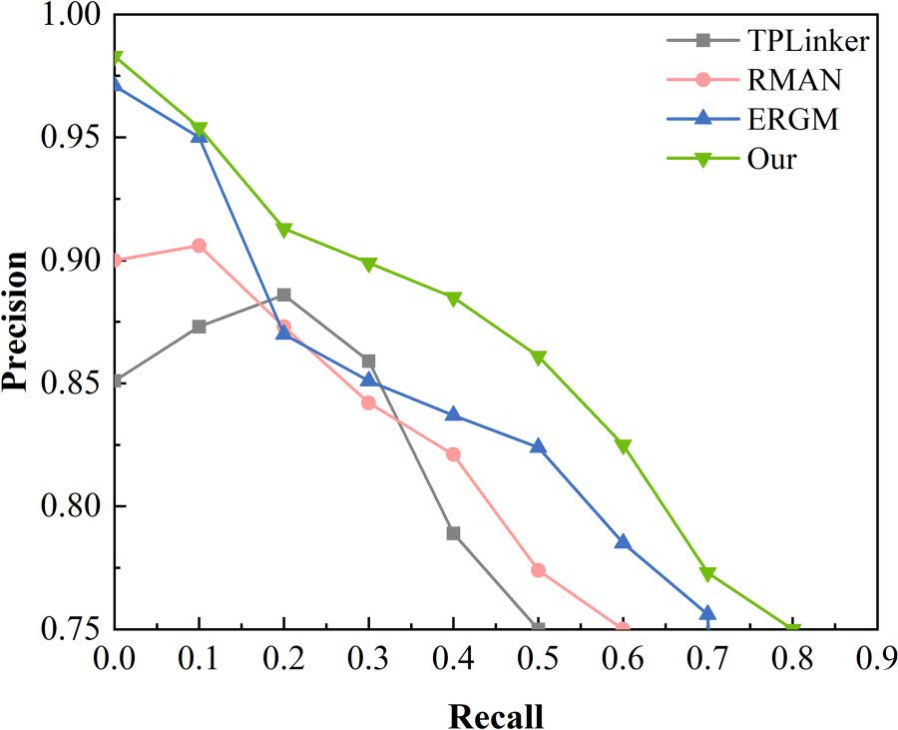
AUC-PR curve.

In order to better evaluate the performance of the model, the AUC-PR curves presented in Fig 6 are trained using the fault dataset of this paper. This makes the DPRM model outperform the other models in Fig 6. The specific experimental results are shown below:

As shown in Fig 6, the DPRM model outperforms the other models in Fig 6. The model proposed in this paper achieves a more competitive accuracy over the entire recall range.The DPRM model performs optimally at all Recall values, especially in the low Recall region, demonstrating extremely high prediction accuracy. Still, its performance decreases at Recall = 0.9. We speculate that this may be due to an increase in the model’s false alarm rate as more positive samples are captured. The ERGM model has the next best performance, with a stable overall performance, but its performance decreases significantly at Recall = 0.8 and above. The RMAN model has a significant decrease in Precision performance at Recall = 0.7 and above, indicating that its high false alarm rate at high recall. The TPLinker model has the worst performance, especially at Recall = 0.6 and above where it fails completely. Overall, the DPRM model performs best in terms of the balance between precision and recall, and the stability shown by DPRM we mainly attribute to the DeBERTa module, which further improves the model’s adaptability to domain-specific data and semantic understanding. This allows our model to excel in the balance of precision and recall. However, further optimisation is still needed in the high Recall region to improve stability, and its stability and generalisation ability can be further improved in the future by adjusting the threshold, optimising the model structure or introducing more training data.

#### 4.2.3 Analysis of overlapping triples.

In order to validate the different overlapping categories this paper categorizes the text into three categories; Normal, SEO, and EPO. The performance performance of the model on these three categories Precision value, Recall value, and F1 value are shown in Tables 6–8. Bold indicates the best result of the experiment. Three models are selected for comparison in this chapter: the ERGM, RMAN, and TPLinker.

**Table 7:** Recall values for the model on Normal, SEO, EPO.

Model	fault datasets		
	Normal	SEO	EPO
TPLinker	0.677	0.468	0.368
RMAN	0.851	0.761	0.683
ERGM	0.868	0.728	**0.780**
**our**	**0.879**	**0.793**	0.760

**Table 8:** F1 values for the model on Normal, SEO, EPO.

Model	fault datasets		
	Normal	SEO	EPO
TPLinker	0.651	0.462	0.462
RMAN	0.848	0.765	0.765
ERGM	0.867	0.730	0.730
**our**	**0.873**	**0.789**	**0.789**

The experimental results demonstrate that the model proposed in this chapter exhibits best performance in both the Normal and SEO categories. In comparison to ERGM, the proposed model demonstrates an improvement in F1 score of 0.6% in the Normal category and 5.9% in the SEO category. However, it is less effective than ERGM in the EPO category, primarily because ERGM employs a negative sampling strategy that effectively mitigates exposure bias, making it more suitable for addressing EPO challenges. In comparison to RMAN and TPLinker, our model demonstrates superior performance in all three categories, particularly in SEO, where it exhibits the most optimal performance among the three models. These enhanced performance outcomes can be attributed to the construction of sentence representations of potential relations facilitated by the relational gated mechanism, which enable the model to retain relevant relational information with greater accuracy. This improvement enhances the sensitivity to relations and effectively addresses the issue of relational overlap. Although the model exhibits clear advantages in handling SEO, its overall performance is somewhat diminished in more complex scenarios. Nevertheless, it remains competitive and demonstrates advantages over baseline models when evaluated.

**Fig 7:**
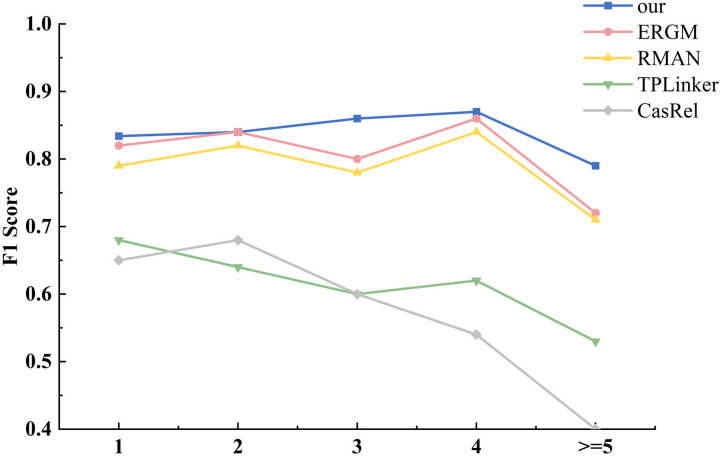
The dataset contains different number of ternary extraction results.

Furthermore, an investigation was conducted to ascertain the model’s capacity for the extraction of multiple triples from the text. The dataset was divided into five categories based on the number of triples present in the sentences. The number of triples present in the sentences ranged from one to five or more. The specific data distributions are detailed in Table 3. As illustrated in Fig 7, the F1 score of the model demonstrated a consistent increase with the growing number of triples in the sentences, indicating the model’s stability and robustness in handling complex text.

### 4.3 Ablation experiment

Ablation experiments were conducted on a fault dataset to assess the impact of each module on the overall performance of the model. The investigation concentrated on the DeBERTa, Multi-Headed Self-Attention, Relational Gated Mechanism, and Entity Pair pairing Module. In this chapter, the complete model is represented by the notation “ALL,” while the performance of the model after the exclusion of a specific module is indicated by the notation “-module.” The results are presented in Table 9 below for your convenience.

**Table 9:** Analysis of ablation experiments.

Model	Precision(%)	Recall(%)	F1 (%)
ALL	**0.863**	**0.868**	**0.872**
-DeBERTa	0.821	0.832	0.831
- Multi-Headed self-Attention	0.846	0.852	0.849
-Relational Gated Mechanism	0.826	0.835	0.829
-Global Entity Pairing	0.848	0.863	0.860

-DeBERTa: Removing the DeBERTa module results in a 4.2% decrease in the F1 score at the sentence level. Due to the large number of name abbreviations for entities in the fault data, DeBERTa results in more accurate identification of certain words, somewhat weakening the boundary ambiguity problem that exists in the co-extraction model.

-Multi-Headed self-Attention: The exclusion of the Multi-Headed Self-Attention (MHSA) mechanism resulted in a 2.3% reduction in the F1 score. This decline suggests that the absence of additional features and semantic information provided by the MHSA mechanism has impact on the model’s ability to encode entity relationships within the fault data.

-Relational Gated Mechanism: The removal of the relational gated mechanism resulted in a 4.3% reduction in the F1 score. This finding demonstrates that relying exclusively on sentence representation is inadequate for addressing the detrimental effects of irrelevant relationships. The incorporation of a relational gating mechanism serves to mitigate the adverse impact of these irrelevant relationships, thereby enhancing the precision of subsequent entity recognition.

-Global Entity Pairing:The removal of the Global Entity Pairing Module and its replacement with BiLSTM resulted in a 1.2% reduction in the F1 score. This reduction indicates that the Global Entity Matching Module reduces the complexity of both the training space and the training time, thereby resulting in significant savings in execution time.

### 4.4 Parameter selection

**Fig 8:**
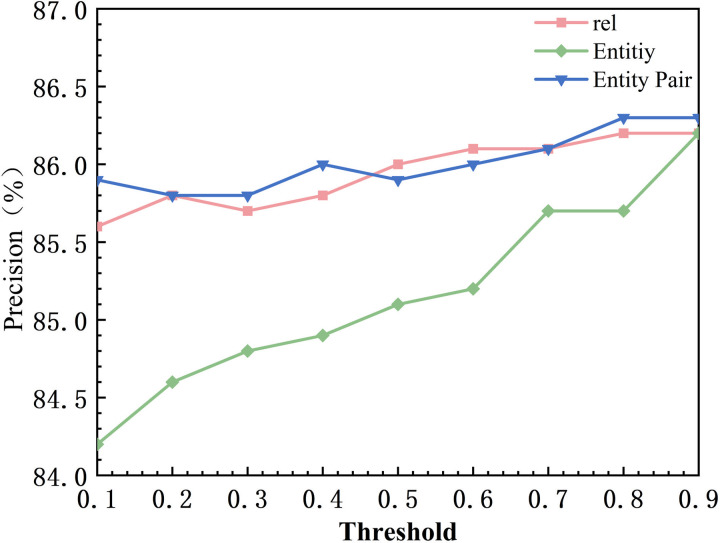
Precision Threshold Comparison.

**Fig 9:**
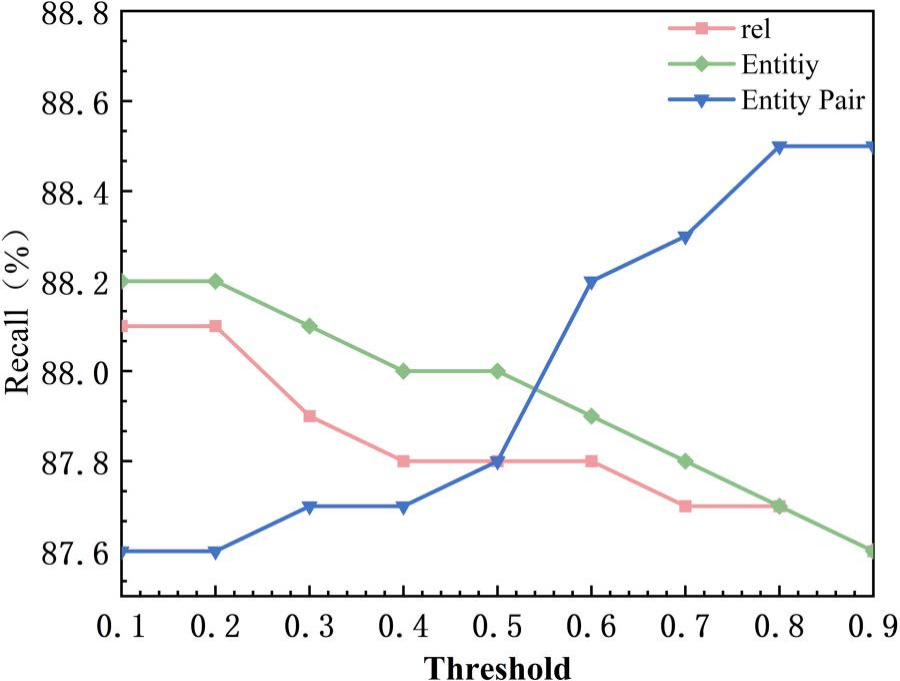
Recall Threshold Comparison.

**Fig 10:**
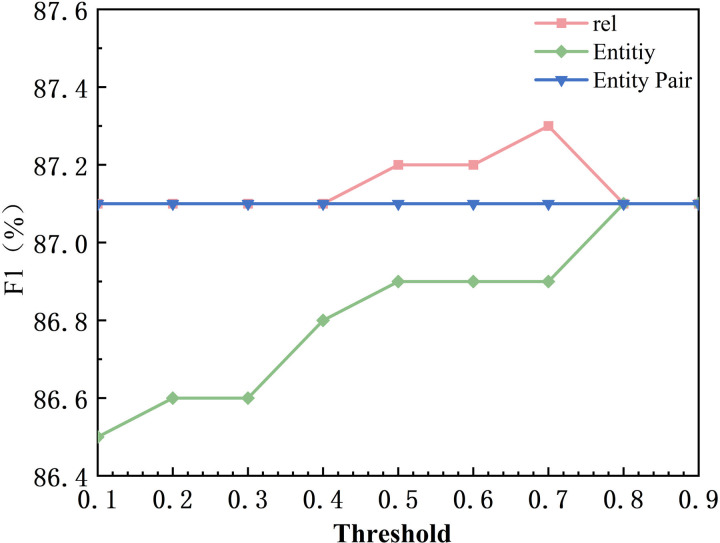
F1Threshold Comparison.

In order to validate the experimental results of the model in three subtasks including potential relationship extraction, entity identification, and, global entity matching under different threshold settings, this chapter performs the threshold experimental parameter analysis in the faulty dataset. The experiment fixes two of the thresholds and performs adjustments to the current thresholds to observe the experimental changes. First of all, the default value of relationship threshold and entity threshold is set to 0.9, and the default value of entity pair threshold is 0.5. The variation range of the thresholds is from 0.1 to 0.9, and the step size is 0.1, and the specific experimental results are shown in Figs 8–10, in which rel represents the model relationship extraction subtask, Entity represents the model real-time recognition subtask. Entity Pair represents the entity pair subtask. Firstly for the relation extraction task, in Fig 8, when the threshold value increases from 0.1 to 0.5, Precision increases from 85.6% to 86%, and when the threshold value increases from 0.5 to 0.9, Precision increases from 86% to 86.2%, and the performance of the model tends to stabilize. This is because the retention of potential relations in the relation extraction makes the model filter out most of the error triples. Whereas the recall value decreases as the relationship threshold increases. When the threshold is increased from 0.1 to 0.9, Recall decreases from 88.1% to 87.6%. This is because higher thresholds may result in some correct but lower confidence ternary groups being filtered out. The F1 score starts to increase at threshold 0.4, reaches a peak at 0.7 (87.3%), and then gradually decreases and remains stable. This suggests that there is an equilibrium point around 0.7 that enables a better trade-off between Precision and Recall. Precision for the entity recognition task is positively correlated with the threshold, as shown in Fig 8, when the threshold is increased from 0.1 to 0.9, Precision increases from 84.2% to 86.2%. This is due to the fact that a higher threshold reduces the misidentification of low-confidence entities. As shown in Fig 9, Recall is negatively correlated with the threshold, and Recall decreases from 88.2% to 87.6% when the threshold is increased from 0.1 to 0.9. This is because a higher threshold may result in some correct but less confident entities being ignored. As shown in Fig 10, the F1 score increases from 0.1 to 0.8 at the threshold and then levels off. This indicates that the higher the threshold, the higher the probability of identifying correct entities in entity recognition. The Precision of the entity pair-matching task positively correlates with the threshold. As shown in Fig 8, Precision increases from 85.9% to 86.3% when the threshold is increased from 0.1 to 0.9. This is due to the fact that the higher threshold filters out more incorrect entity pairs with low confidence. In Fig 9, for entity pairs with thresholds from 0.1 to 0.8, Recall increases from 87.6% to 88.5% and stabilizes between 0.8 and 0.9. Its magnitude is proportional to the recall rate, which may be due to the fact that the entity pair matching task itself is difficult with many negative samples with high confidence. In Fig 10, when the thresholds for entities and relations are fixed at 0.9, the F1 value is not affected by the adjustment of the entity pair thresholds. It is hypothesized that this may be due to the fact that more relationships and entities are accurately identified in the preorder task, and therefore in the final entity pair matching task, the entity pairs are positive samples in most cases, which improves the confidence of the model.

In terms of experimental parameters, the overall performance of the model was optimized by the training comparison of the model by setting the dimension of Bi-GRU to 300, the number of self-attentive heads to 12, and Dropout to 0.3. This finding emphasizes the importance of parameter selection for model performance. In this paper, we train the model by adjusting the learning rate and Epoch. The learning rate and epoch settings are crucial for model training. The learning rate directly affects the updating speed and convergence stability of the model weights. A high learning rate may cause the model to diverge from the optimal solution quickly, while a low learning rate may cause the weights to be updated slowly and prolong the training period, and the number of Epochs is related to the model’s ability to learn the features of the data adequately and to generalize the model. Insufficient Epoch may cause the model to fail to learn sufficiently, while too much Epoch may cause overfitting.

**Fig 11:**
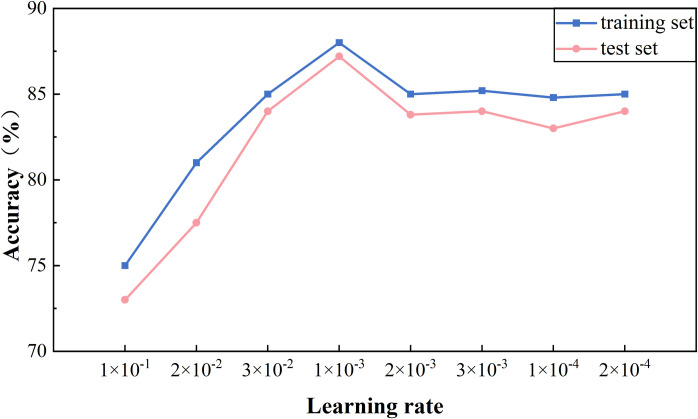
Changes in the impact of accuracy.

**Fig 12:**
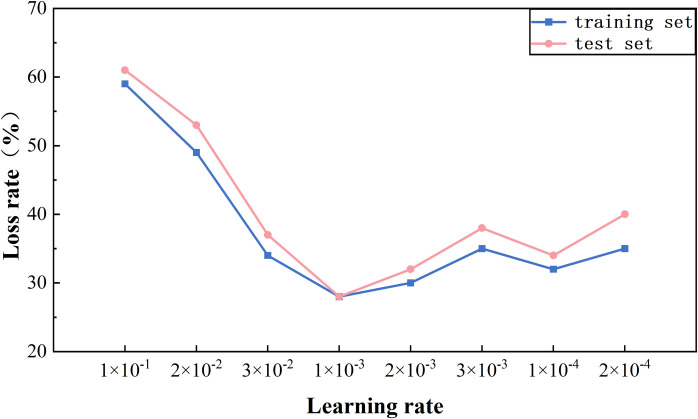
Changes in the impact of the rate of loss.

Since DBM is used in the joint extraction model for semantic representation, the setting of different learning rates is particularly important in influencing the extraction performance of the subsequent model. In the experiment, different learning rates are set to compare the F1 score of the model with the loss value. As shown in Fig 11, different learning rates are set during training and testing to compare the accuracy of the model in extracting ternary groups. As shown in Fig 12, different learning rates are set for the model during training and testing to see the changes in the loss rate of the model. The comparison experiment shows that the model has a higher accuracy rate in the training set when the learning rate is 1 × 10^−3^ and 3 × 10^−3^, but a lower accuracy rate of 3 × 10^−3^ in the testing set. As can be seen in Fig 12, the loss rate of the model at learning rates of 1 × 10^−3^ and 2 × 10^−3^ is lower compared to the loss rate of the other learning rates, and the difference between the loss rate of the training set and the test set is very small in the model with a learning rate of 1 × 10^−3^. Therefore, the learning rate of 1 × 10^−3^ is the best choice for the model considering the high accuracy and low loss rate of the model. This not only ensures the high accuracy of the model on the training set but also maintains the stability of the model on the test set, thus achieving the generalization ability of the model.

**Fig 13:**
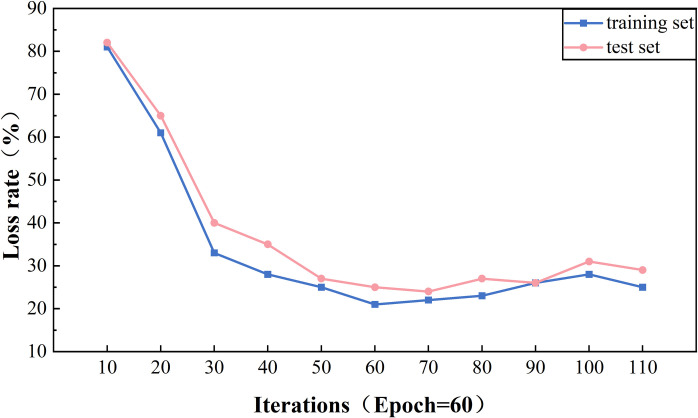
Changes in the impact of the number of model iterations on the accuracy rate.

Fig 13 illustrates the effect of different Epoch on the accuracy rate of the model on the training and test sets. As can be observed from the trend of the folds in the figure, the model achieves the highest accuracy rate on the training set when Epoch reaches 60 and 70. However, it is worth noting that at Epoch = 70, there is a drop in the accuracy rate of the model on the test set, which may imply that the model is starting to show a slight overfitting phenomenon. From Epoch = 60 onwards, the change in accuracy rate stabilizes, suggesting that the model is approaching the saturation point of its performance.

**Fig 14:**
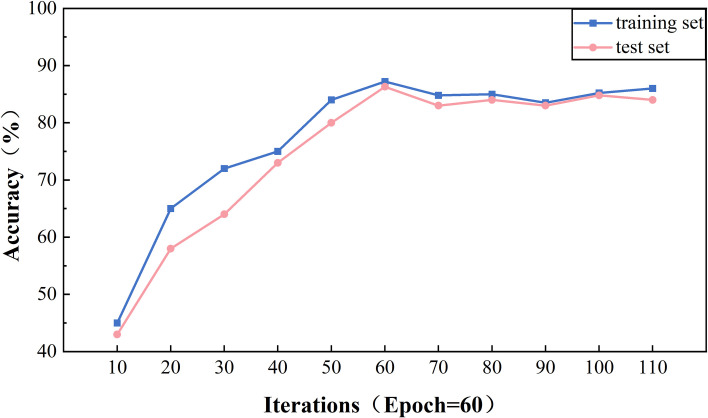
Changes in the impact of the number of model iterations on the loss rate.

Fig 14 further reveals the effect of different numbers of iterations on the loss rate of the model’s training and validation sets. At the early stage of Epoch, i.e., before Epoch = 30, the loss rate of the model shows a significant decrease, which indicates that the model is learning the features of the data quickly. As Epoch increases, especially at Epoch = 70 and Epoch = 60, the model’s loss value reaches its lowest point, which is consistent with the trend of the accuracy rate. As Epoch exceeds 50, the loss rate begins to level off, further confirming the stabilization of the model performance.

Considering the performance of the accuracy rate and the loss rate together, we can conclude that the parameter configuration of the model reaches its optimal state at Epoch = 60. This configuration not only achieves a low loss rate on the training set but also maintains a high accuracy rate on the test set, thus ensuring the model’s ability to generalize, helping to avoid overfitting, and ensuring that the model can fully learn the features of the data.

## 5 Conclusion

This chapter puts forth a DeBERTa-based joint extraction model (DPRM). This model effectively addresses the issue of relationship overlap within the manufacturing fault domain while adapting to the specific characteristics of the task. The model is comprised of three modules: the semantic representation module, the relationship extraction and entity recognition module, and the global entity pairing module. It is noteworthy that the semantic representation module employs a DeBERTa pre-trained encoder for the first time in the manufacturing fault domain, thereby mitigating the entity boundary ambiguity issues commonly encountered in joint extraction models. Furthermore, a multi-headed self-attention mechanism is employed to capture the semantic information among the attention words. The relationship extraction and entity recognition module serves to identify potential relations within sentences, thereby reducing the likelihood of irrelevant relationships being identified. The introduction of the relational gated mechanism enables the model to refine the selection of faulty relation information, thereby enhancing its expressive capability. The global entity pairing module simultaneously extracts all entity pair information, thereby reducing both the space and time complexity of training. The experimental results on a fault dataset validate the effectiveness of the model and demonstrate improvements in accuracy. Nevertheless, the model persists in exhibiting exposure bias. Future work will concentrate on investigating further techniques to overcome these existing challenges and further enhance the model’s accuracy. Specifically, we plan to start from the following aspects:

1. Curriculum Learning: by gradually increasing the complexity of the training data, the model can be better adapted to complex patterns, thus reducing the impact of exposure bias. For example, you can start training with simple samples and gradually transition to more complex samples.
2. Adversarial Training: Adversarial sample generation techniques are introduced to enhance the robustness of the model by generating challenging samples. This helps the model to better handle patterns not seen in the training data.
3. Self-supervised Learning: Use self-supervised learning methods to pre-train the model so that it can learn more semantic information without large amounts of labeled data. This reduces the reliance on labeled data, thus reducing exposure bias.
4. Data Augmentation: data augmentation techniques are used to generate more training samples, especially those patterns that occur less frequently in existing datasets. This helps the model to generalize better to different scenarios.
5. Model Ensemble: Combine the predictions of multiple models to reduce the bias of individual models by integrating learning methods. This improves the stability and accuracy of the model.

Through these specific techniques and methods, we hope to effectively overcome the exposure bias problem and further improve the performance of the model. Future research will delve into the feasibility of these directions and validate them in practical applications.
